# The Mediating Role of Loneliness Between Social Support and Depressive Symptoms Among Chinese Rural Adolescents During COVID-19 Outbreak: A Comparative Study Between Left-Behind and Non-left-behind Students

**DOI:** 10.3389/fpsyt.2021.740094

**Published:** 2021-08-23

**Authors:** Tianya Hou, Yawei Xie, Xiaofei Mao, Ying Liu, Jianguo Zhang, Jing Wen, Yan Chen, Zhechao Luo, Wenpeng Cai

**Affiliations:** ^1^Faculty of Psychology, Second Military Medical University, Shanghai, China; ^2^College of Basic Medical Sciences, Second Military Medical University, Shanghai, China

**Keywords:** depression, perceived social support, loneliness, left-behind students, non-left-behind students, COVID-19

## Abstract

**Introduction:** The COVID-19 pandemic has greatly impacted people's life across the globe. In a public health crisis, rural adolescents are more prone to mental health problems. The current study aimed to investigate the prevalence of depressive symptoms among Chinese rural adolescents during the COVID-19 outbreak, and examine the association between perceived social support and depressive symptoms and its underlying mechanisms.

**Method:** Perceived Social Support Scale, UCLA Loneliness Scale, Patient Health Questionnaire-9 were administrated to 826 rural adolescents from Anhui Province, China, amid the COVID-19 crisis. Mackinnon's four-step procedure was employed to examine the mediating effect, while Hayes PROCESS macro was utilized to test the moderated mediation model.

**Results:** The results showed the rate of depressive symptoms among rural adolescents in China was 77.6% during the outbreak of COVID-19. Female left-behind students and non-left-behind students from disrupted families experienced more depressive symptoms (all *P* < 0.05). Loneliness mediated the association between perceive social support and depressive symptoms and the indirect effect was stronger in left-behind adolescents in comparison to non-left-behind adolescents during the COVID-19 pandemic.

**Conclusion:** Depressive symptoms are extremely prevalent among Chinese rural adolescents during the COVID-19 outbreak, and perceived social support plays a protective role against depressive symptoms. Chinese rural adolescents, especially left-behind students, could benefit from the interventions aimed at enhancing the perceived social support and reducing loneliness during the COVID-19 pandemic.

## Introduction

The outbreak of COVID-19 has brought great challenges for both physical and mental healthcare around the world and disrupted daily life for everyone (1). At present, the outbreak still rages in many regions of the world (2). As of June 10, 2021, COVID-19 has led to more than 3 million confirmed deaths out of 173 million confirmed cases (3). COVID-related stressors, such as enhanced social isolation, decreased prosocial activities, reduced access to mental health services, increased concerns over health, intensified family conflict, were found to be related to higher levels of psychological problems (4). Public health measures to curb the spread of COVID-19 have exerted adverse impacts on the mental health, especially for adolescents. Adolescence is a critical phase for the formation of identity (5). Compared with adults, adolescents are more likely to experience intense emotions with greater frequency and fluctuation (6).

Perceived social support refers to an individual's cognitive appraisal of the quality and quantity of social connections. Evidence suggested that social and community ties played a critical role in the etiology of disease through the underlying psychological and physiologic mechanisms (7). There is a long history in psychological studies which has investigated the association between perceived social support and mental health outcomes. Evidence suggested the protective effect of perceived social support on mental health (8, 9). Previous literature found students with lower levels of social support were six times more likely to experience symptoms of depression than those with higher levels of social support (9). Recently, a substantial body of research has investigated the role of perceived social support during the COVID-19 pandemic (10). Negative associations between perceived social support and depressive symptoms amid the COVID-19 outbreak have been documented (10, 11). Hence, we speculated that perceived social support was negatively associated with depressive symptoms among rural students during COVID-19 pandemic.

Loneliness, also termed perceived social isolation (12), is a common risk factor of depressive symptoms. Loneliness is defined as the disparity between one's desired and actual levels of social relationships (13). Perceived social isolation in humans is associated with enhanced sympathetic tonus, increased HPA activation, reduced inflammatory control and decreased expression of genes regulating glucocorticoid responses, which might further lead to deleterious health outcomes (14). Loneliness is suggested to be a precursor of depression (15, 16). In addition, research evidence also suggested perceived social support could reduce the feeling of loneliness experienced by adolescents (17). Moreover, in a recent study conducted by Liang et al. (18), loneliness partially mediated the association between perceived social support and depressive symptoms among Chinese rural-to-urban migrants. Therefore, we expected that loneliness was not only directly associated with symptoms of depression, but also played a mediation role in the association between social support and depressive symptoms among rural adolescents.

Although perceived social support could influence loneliness among adolescents, not all adolescents with lower levels of perceived social support report higher levels of loneliness. Therefore, it is of great importance to explore the influential factor that might moderate the relationship between social support and loneliness. Over the last three decades, the rapid urbanization of China has led to a large scale of domestic migration (19). Due to the fast development of public transportation systems, rural migrant workers could move from rural to urban areas at relatively low cost and easily stay connected with their sending societies (20). A large number of rural residents migrated from rural to urban settings for better employment opportunities and higher salaries, leaving their children in their hometown on account of high living expenses and huge obstacles to health care and education in urban regions (21). Rapid population mobility has placed tremendous burdens on migrants and their families (22). As the product of this phenomenon, “left-behind children” has aroused great concern. Left-behind students refer to underage students who stayed at rural areas while one or both of their parents leave to work in cities for at least 6 months (23). Non-left-behind students might have experienced more parental supervision and more actual parental company (24), which might reduce the effect of perceived social support on loneliness. Therefore, we hypothesized that the association between perceived social support and loneliness would be strengthened for left-behind students in rural China during COVID-19 pandemic.

**Figure 1:**
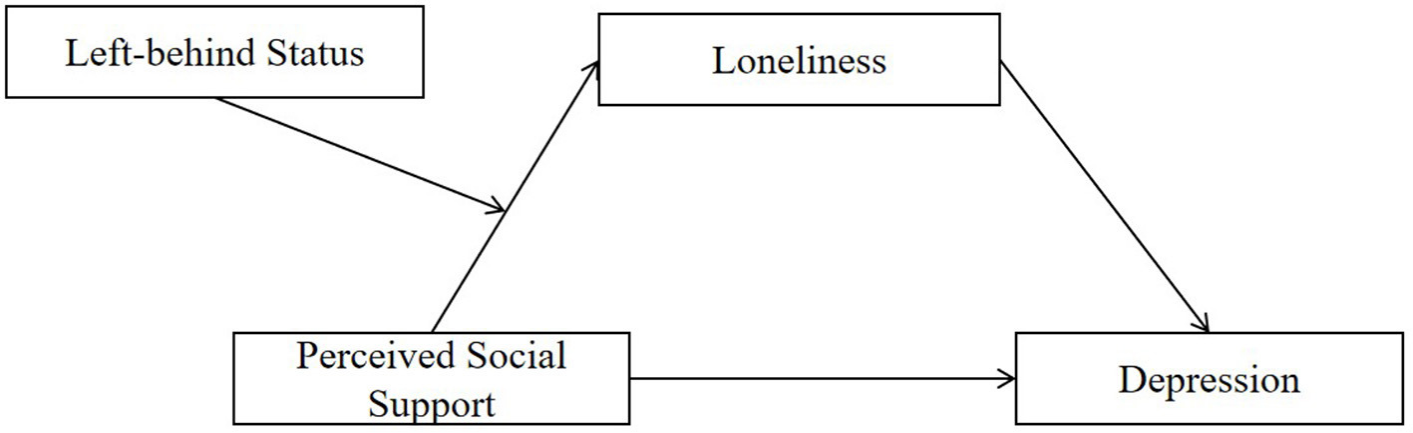
Conceptual model.

To date, there is an absence of the studies on the potential mechanisms underlying the association between social support and depressive symptoms among rural Chinese adolescents during COVID-19 pandemic. Therefore, the present study aimed to investigate (a) the prevalence of depressive symptoms among rural adolescents during COVID-19 pandemic, (b) whether perceived social support is negatively associated with depressive symptoms, (c) whether loneliness mediates the association between perceived social support and depressive symptoms, and (d) whether the path between perceived social support and loneliness differs between left-behind students and non-left-behind students in rural China during COVID-19 epidemic. A moderated mediation model (see Figure 1) is constructed to address the hypotheses that the effect of perceived social support on depressive symptoms was mediated by loneliness and moderated by left-behind status. Specifically, the relationship between perceived social support and loneliness would be more powerful in left-behind students than non-left-behind students.

## Methods

### Participants and Procedures

The cross-sectional study was conducted from 28 April to 1 May, 2021 in rural regions of Anhui province, China, which is a one of the principal migrant-sending areas of China (25). A random cluster sampling was employed to obtain a sample of rural students from 15 classes in five senior high schools. The inclusion criteria were (a) age ≤ 18 years; (b) no cognitive impairment or/and dyslexia and (c) being born and raised in a rural setting. The exclusion criterion was set for subjects who were previously diagnosed with psychiatric illness. A total of 831 rural students were invited to take part in the study, with one student refusing to answer the questionnaire and 4 students returning incomplete questionnaire. Finally, a total of 826 rural students were included in the analysis (effective response rate 99.4%).

This research was approved by the Research Ethics Commission of Navy Medical University. The participants aged 18 years or the parent or legal guardian of the participants aged under 18 years provided written informed consent before the participants anonymously completed the survey in the classroom. All participants were free to withdraw from the study at any time.

### Measures

#### Demographics

The socio-demographic variables included age (≤ 16 or >16), sex (male and female), parental highest educational attainment (junior middle school or below and high school or above), family structure (intact family and disrupted family), perceived socioeconomic status (blow average and average or above) (26, 27), only-child status (whether they were the only child in their families) and left-behind status (whether one of the parents or both parents migrated to work in cities for at least 6 months). Students from disrupted family referred to students whose parents separated, divorced, deceased or never married (28). Students from intact family was defined as those with both biological parents present and married (29, 30).

#### Social Support

The Chinese version of Perceived Social Support Scale (PSSS) is a 12-item self-report measure assessing the perception of social support from three domains (31): family (i.e., “I can talk about my problems with my family”), friends (i.e., “I have friends with whom I can share my joys and sorrows”) and significant other (i.e., “There is a special person who is around when I am in need”). Each item is scored on a 7-point Likert scale, ranging from 1 (strongly disagree) to 7(strongly agree). The total score is obtained by summing the scores of all items and ranged from 12 to 84 with the higher scores denoting higher levels of perceived social support. The scale has demonstrated satisfactory reliability and validity and been successfully used in adolescents (32, 33). In the present study, the Cronbach's alpha was 0.921, 0.857, 0.841, and 0.815 for total scale, family subscale, friend subscale and significant other subscale.

#### Loneliness

The 20-item UCLA Loneliness Scale was used to evaluate the feelings of loneliness. Subjects were asked to rate each item on a 4-point Likert-type scale from “0” (I never feel this way) to “3” (I often feel this way). All 20 items were summed up to create a total score ranging from 0 to 60, with higher scores indicating higher levels of loneliness. The measure has been widely used among Chinese adolescents with demonstrated psychometric properties (34). In the present study, Cronbach's alpha was 0.833.

#### Depressive Symptoms

Depressive symptoms were measured by Patient Health Questionnaire-9 (PHQ-9). Each item is answered on a 4-point Likert scale ranging from “0” (none) to “3” (almost every day). The range for the scale is between 0 and 27, with a higher score denoting greater depressive symptoms. The cutoff score for detecting depressive symptoms was 5 (35, 36). The scale has been used extensively among adolescents and shown adequate reliability and validity (37, 38). In the current study, Cronbach's alpha was 0.855.

### Statistical Analysis

Firstly, common method bias was examined using Harman single factor test and descriptive analyses were calculated to describe the sociodemographic characteristics stratified by left-behind status. Secondly, linear regressions were performed to calculate the univariate association between demographic variables and greater depressive symptoms stratified by left-behind status and Pearson's correlation analyses were conducted to investigate the bivariate correlations between the variables of interest. Thirdly, the mediation effect was tested by MacKinnon's four-step procedure (39). Four specific requirements should be met: (1) a significant association between perceive social support and greater depressive symptoms; (2) a significant association of perceived social support with loneliness; (3) a significant association between loneliness and greater depressive symptoms when perceived social support was controlled; (4) the significant coefficient of indirect pathway between perceived social support and greater depressive symptoms via loneliness. Bias-corrected percentile bootstrap method was used to determine the last condition, producing a 95% bias-corrected confidence interval (CI) from 5,000 resamples. Hayes (40) PROCESS macro (Model 4) was employed to estimate the parameter. Finally, Hayes (40) PROCESS macro (Model 7) was utilized to examine the moderated mediation model, followed by simple slope test.

Age, sex, parental highest educational attainment, perceived family economic status, family structure and only-child status were included in all models as potential confounders. We standardized all variables before modeling. Statistical analyses were conducted using SPSS version 26. Statistical significance was considered as *p* < 0.05 (two-tails).

## Results

### Common Method Bias Test

Given that the research data were obtained by self-report using an online questionnaire, Harman single factor was employed to examine whether common method bias would be a potential validity threat (2, 41). The KMO value was 0.92 (*p* < 0.001), which indicated the research data were suitable for factor analysis. There were seven values with eigenvalue more than 1 and the first factor presented a variance of 23.786%, which did not reach the criterion of 40%. Therefore, the results suggested common method bias was not a serious problem in the study.

### Descriptive Statistics Stratified by Left-Behind Status

Table 1 presented the demographic characteristics of the left-behind students and non-left-behind students. Among 826 adolescents, 591 (71.5%) were left-behind students. Most participants were aged between 17 and 18 years (77.2% in left-behind students, 74.5% in non-left-behind students), were male (57.4% in left-behind students, 56.6% in non-left-behind students), reported a parental highest educational level of junior middle school or below (77.5% in left-behind students, 76.3% in non-left-behind students), perceived family economic status to be average or above average (81.7% in left-behind students, 76.6% in non-left-behind students), were from intact family (90.7% in left-behind students, 91.9% in non-left-behind students), and were not the only child in their family (90.0% in left-behind students, 91.5% in non-left-behind students).

**Table 1:** The demographic characteristics of the left-behind students and non-left-behind students (N = 826).

**Variables**	**Total** **(** ***n*** **= 826)**	**Left-behind students** **(** ***n*** **= 591)**	**Non-left-behind students** **(** ***n*** **= 235)**
	No. (%)	No. (%)	No. (%)
Total	826 (100)	591 (71.5)	235 (28.5)
**Age (years)**			
≤ 16	195 (23.6)	135 (22.8)	60 (25.5)
>16	631 (76.4)	456 (77.2)	175 (74.5)
**Sex**			
Male	472 (57.1)	339 (57.4)	133 (56.6)
Female	354 (42.9)	252 (42.6)	102 (43.4)
**Parental highest educational attainment**			
Junior middle school or below	631 (76.4)	458 (77.5)	173 (73.6)
High school or above	195 (23.6)	133 (22.5)	62 (26.4)
**Perceived family economic status**			
Below average	163 (19.7)	108 (18.3)	55 (23.4)
Average/above average	663 (80.3)	483 (81.7)	180 (76.6)
**Family structure**			
Intact family	752 (91.0)	536 (90.7)	216 (91.9)
Disrupted family	74 (9.0)	55 (9.3)	19 (8.1)
**Only child**			
Yes	79 (9.6)	59 (10.0)	20 (8.5)
No	747 (90.4)	532 (90.0)	215 (91.5)

### Associations of Demographic Variables With Depressive Symptoms

The prevalence of depressive symptoms was 77.6% among Chinese rural students during COVID-19 pandemic. The prevalence of depressive symptoms was 77.7% among left-behind students, while the rate of depressive symptoms was 77.4% among non-left-behind students. The results of the univariate logistic regression (see Table 2) showed that across the overall sample, female adolescents were more likely to experience depressive symptoms than their male counterparts (β = 2.346, 95% CI = [0.132, 1.483]). Nevertheless, the difference disappeared in non-left-behind students. In the overall sample, students from disrupted family reported more symptoms of depression than those from intact family (β = 2.224, 95% CI = [0.156, 2.497]). However, the disparity was not significant in left-behind students.

**Table 2:** Association of demographic characteristics with depressive symptoms stratified by left-behind status (*N* = 826).

**Variables**	**Total sample (** ***n*** **= 826)**	**Left-behind students (** ***n*** **= 591)**	**Non-left-behind students (** ***n*** **= 235)**
	**β (95% CI)**	**β (95% CI)**	**β (95% CI)**
**Age (years)**			
≤ 16	Reference	Reference	Reference
>16	−1.701 (−1.475, 0.101)	−1.629 (−1.723, 0.161)	−0.688 (−1.959, 0.945)
**Sex**			
Male	Reference	Reference	Reference
Female	2.346 ^*^ (0.132, 1.483)	2.153 ^*^ (0.077, 1.673)	0.997 (−0.630, 1.922)
**Parental highest educational attainment**			
Junior middle school or below	Reference	Reference	Reference
High school or above	−0.913 (−1.156, 0.422)	−0.866 (−1.367, 0.530)	−0.274 (−1.638, 1.237)
**Perceived family economic status**			
Below average	Reference	Reference	Reference
Average/Above average	−1.755 (−1.593, 0.089)	−1.162 (−1.630, 0.418)	−1.510 (−2.631, 0.348)
**Family structure**			
Intact family	Reference	Reference	Reference
Disrupted family	2.224^*^ (0.156, 2.497)	1.344 (−0.430, 2.294)	2.062^*^ (0.107, 4.715)
**Only child**			
Yes	Reference	Reference	Reference
No	0.951 (−0.588, 1.691)	0.492 (−0.991, 1.653)	1.083 (−1.020, 3.511)

* *P < 0.05*.

### Bivariate Analysis

Table 3 presented the descriptive statistics and correlations among variables of interest. The results showed that perceived social support and all three subscales were all significantly and negatively correlated with loneliness and depression (all *P* < 0.001). Loneliness was significantly and negatively related to depression (*P* < 0.001).

**Table 3:** Descriptive statistics and correlations among variables (*N* = 826).

	**Mean (SD)**	**1**	**2**	**3**	**4**	**5**	**6**
1. Left-behind status	1.28 (0.45)						
2. Friend subscale of PSSS	17.29 (5.28)	0.011					
3. Family subscale of PSSS	17.01 (4.91)	−0.015	0.615^***^				
4. Significant others subscale of PSSS	17.09 (4.91)	−0.029	0.696^***^	0.763^***^			
5. Perceived social support (PSSS)	51.38(13.45)	−0.012	0.871^***^	0.885^***^	0.917^***^		
6. Loneliness	47.92 (8.55)	−0.042	−0.381^***^	−0.477^***^	−0.459^***^	−0.459^***^	
7. Depression	8.16 (4.91)	−0.034	−0.297^***^	−0.296^***^	−0.296^***^	−0.296^***^	0.425^***^

*** *P < 0.001*.

### Analysis of Loneliness as a Mediator

To examine the mediating effect of loneliness, MacKinnon's (39) four-step procedure was performed. Firstly, perceived social support was significantly associated with depressive symptoms (β = −0.327, *P* < 0.001) (see Model 1 in Table 4). Secondly, perceived social support was significantly related to loneliness (β = −0.487, *P* < 0.001) (see Model 2 in Table 4). Thirdly, loneliness was significantly associated with depressive symptoms after controlling for perceived social support (β = 0.337, *P* < 0.001) (see Model 3 in Table 4). Finally, the result of the biased-corrected percentile bootstrap method suggested the indirect effect of perceived social support on depressive symptoms through loneliness was significant since the 95% CI does not include zero (ab = −0.164, SE = 0.022, 95% CI = [−0.209, −0.122]). The mediation effect accounted for 49.76% of the total effect. Therefore, all four requirements for the mediating role of loneliness were met and loneliness mediated the impact of perceived social support on depressive symptoms.

**Table 4:** Mediation analysis (*N* = 826).

	**Model 1(Depression)**		**Model 2 (Loneliness)**		**Model 3(Depression)**		**Indirect effect of loneliness**				
	**β**	***t***	**β**	***t***	**β**	***t***		**Indirect effect**	**SE**	**LLCI**	**ULCI**
Perceived social support	−0.327^***^	−9.934	−0.487^***^	−16.050	−0.163^***^	−4.533	Loneliness	−0.164	0.022	−0.209	−0.122
Loneliness					0.337^***^	9.353					
${\text{R}}_{\text{adj}}^{2}$	0.125		0.257		0.210						
*F*	16.751^***^		40.314^***^		27.140^***^						

*All models are adjusted for age, sex, parental highest educational attainment, perceived family economic status, family structure, and only-child status*.

*** *P < 0.001*.

### Testing for Moderated Mediation

The present study hypothesized that left-behind status would moderate the impact of perceived social support on loneliness. The results presented that the interaction between perceived social support and loneliness had a significant effect on depression (β = 0.174, *P* < 0.01), indicating the effect of perceived social support on loneliness was moderated by left-behind status (see Table 5). Thus, the moderated mediation model was established as the first stage of the mediation effect was moderated by left-behind status.

**Table 5:** Testing the moderated mediation effect (*N* = 826).

	**β**	***SE***	**LLCI**	**ULCI**
**Mediator variable model (Outcome: Loneliness)**				
Perceived social support	−0.492^***^	0.030	−0.552	−0.433
Left-behind status	−0.104	0.067	−0.235	0.027
Perceived social support × Left-behind status	0.174^**^	0.065	0.047	0.301
**Dependent variable model (Outcome: Depression)**				
Perceived social support	−0.163^***^	0.036	−0.233	−0.092
loneliness	0.337^***^	0.036	0.267	0.408
**Conditional indirect effect analysis**				
Left-behind students	−0.183	0.025	−0.233	−0.136
Non-left-behind students	−0.124	0.025	−0.178	−0.080
Index of moderated mediation	0.059	0.025	0.010	0.106

*All models are adjusted for age, sex, parental highest educational attainment, perceived family economic status, family structure, and only-child status*.

** *P < 0.01*.

*** *P < 0.001*.

**Figure 2:**
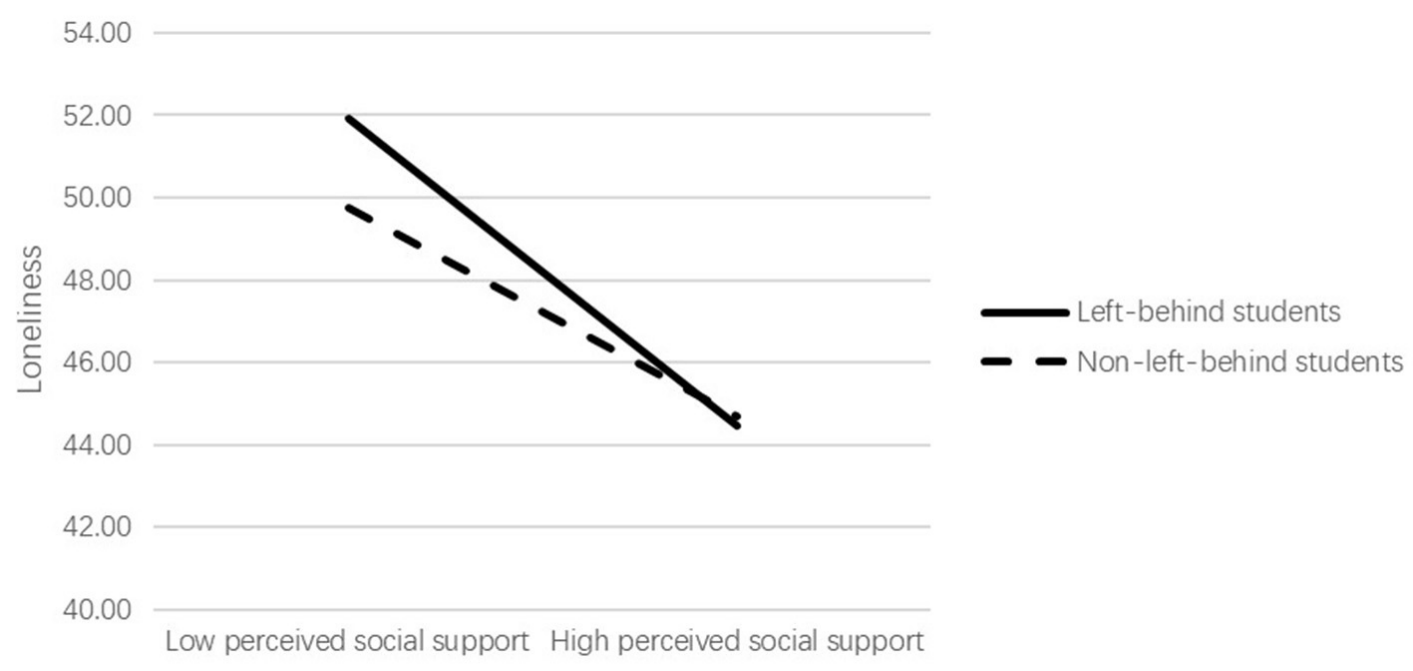
Left-behind status as a moderator of the association between perceived social support and loneliness.

The results of the simple slope analysis showed that perceived social support was significantly and negatively associated with loneliness for left-behind students (β_simple_ = −0.542, *P* < 0.001), while for non-left-behind students, the association between perceived social support and loneliness was still significant, but much weaker (β_simple_ = −0.369, *P* < 0.001). For descriptive purposes, the current study plotted the association of perceived social support with loneliness, separately for left-behind and non-left-behind adolescents (see Figure 2).

Table 5 also showed the conditional indirect effect of perceived social support on depression. The 95% CI that does not contain zero suggested the establish of moderated mediation model. Specifically, the indirect effect of perceived social support on depression via loneliness was stronger for left-behind students (β = −0.183, 95% CI = [−0.233, −0.136]) in comparison to non-left-behind students (β = −0.124, 95% CI = [−0.178, −0.080]).

## Discussion

The results presented that the overall prevalence of depressive symptoms among rural adolescents in China was 77.6% during COVID-19 epidemic, which is much higher than the previously-reported prevalence among Chinese adolescents (11.8–57.0%) during COVID-19 pandemic (42, 43). In addition, this is also higher than the existing range of the prevalence of depressive symptoms (12.1–51.4%) among rural adolescents in the non-epidemic period (44, 45). In line with the previous literature (46), the difference in the prevalence of depressive symptoms between left-behind students and non-left-behind students was not significant. Thus, depressive symptoms are very common among both left-behind and non-left-behind students during COVID-19 pandemic because of the unpleasant experiences during the COVID-19 pandemic such as worry about infection, lack of interpersonal contact with friends, limited private space at home and frequent conflict with family members (43).

Our results presented that females reported higher levels of depressive symptoms only among left-behind students. According to ABC model, the sex differences of depression could be attributed to the affective (emotional stability), biological (genetics, pubertal hormones, and neural development) and cognitive (cognitive styles, rumination, and objectified body consciousness) factors (47, 48). Evidence from the previous literature (49, 50) suggested that left-behind students were more likely to use negative coping styles and have emotional problems, which might partially explain why sex differences were only observed in left-behind students. Our findings also showed that family structure was associated with depressive symptoms only among non-left-behind students. Compared with non-left-behind students from intact families, those from disrupted families were more likely to experience depressive symptoms, which is consistent with the previous literature (51). Parental divorce is associated with less parental care and an elevated risk of emotional and behavioral problems (52, 53) such as social problems, withdrawal, juvenile delinquency, which would make them more susceptible to psychological symptoms. For left-behind students from both intact and disrupted families, they do not live with both parents and might lack parental care since at least one of their parents migrates to work (54). Hence, no significant effect of family structure on depressive symptoms was observed among left-behind students.

As hypothesized, loneliness partially mediated the association between perceived social support and depressive symptoms among rural adolescents during COVID-19 pandemic, which was consistent with the previous literature (17, 18, 55). According to the theory of mental incongruity, loneliness occurs when individuals perceived a difference between their expected and actual levels of social support (56). Thus, rural adolescents with lower levels of perceived social support might be more likely to feel loneliness, which would further result in depressive symptoms. In addition, the results of the moderated mediation model also revealed that the left-behind status moderated the indirect impact of perceived social support on depression through loneliness among rural adolescents during the pandemic. Specifically, the association between perceived social support and loneliness was stronger for left-behind students in comparison to non-left-behind students. This might be attributed to the absence of parental care among left-behind students, including physical companionship, parental supervision and parental guidance (57), which might contribute to the strengthened association between perceived social support and loneliness among left-behind adolescents.

The current study has profound implications both theoretically and practically. In theory, our findings shed insight into the underlying mechanisms linking perceived social support to depressive symptoms among left-behind adolescents during the COVID-19 outbreak. In practice, our results could inform healthcare professionals to develop more targeted interventions for preventing depressive symptoms in rural adolescents amid the COVID-19 crisis. Programs to enhance perceived social support may be an important aspect of depression prevention for rural students. Moreover, interventions for alleviating loneliness, such as mediation, mindfulness and social cognitive skills training (58), should also be designed and prioritized for left-behind students.

Several limitations need to be noted when evaluating the findings of our study. Firstly, the study design of the current research is cross-sectional, indicating the causality could not be established since the data only represent a given point in time. Hence, further longitudinal studies should be conducted to validate the results. Secondly, self-reported measurements were employed in the study, which might result in self-report biases. Further research could collect data from multi-informants (i.e., classmates and teachers). Thirdly, students in our study sample were only from Anhui province in China, which might limit the generalization of our results. Currently, our results could generalize to rural students in Anhui province. In the future studies, multicenter approaches are recommended to recruit participants. Finally, depressive symptoms of students in rural China could be influenced by many other variables. The model in the present study could only explain a part of the variance. Therefore, further research is suggested to incorporate more factors for a more comprehensive understanding of depressive symptoms among rural students.

As far as we know, this is the first study reporting the prevalence of depressive symptoms among rural adolescents during COVID-19 pandemic and investigating the potential mechanisms underlying the association between perceived social support and depression among this specific group. Depressive symptoms are prevalent in 77.6% of rural adolescent during the COVID-19 pandemic. Healthcare professionals and the government should pay special attention to rural adolescent, especially female left-behind students and non-left-behind students from disrupted families. Rural adolescents with lower levels of perceived social support, especially left-behind students, could benefit from the interventions aimed at enhancing the perceived social support and reducing loneliness.

## Data Availability Statement

The raw data supporting the conclusions of this article will be made available by the authors, without undue reservation.

## Ethics Statement

The studies involving human participants were reviewed and approved by Second Military Medical University. Written informed consent to participate in this study was provided by the participants' legal guardian/next of kin.

## Author Contributions

TH, YX, and XM contributed to the writing of this article and the statistical analysis and are the co-first authors. WC organized the whole study, including carrying out this study, putting forward the study, and were the corresponding authors. YL, JW, YC, and ZL contributed to the design and statistical analysis. All authors contributed for editing the article and have approved the final manuscript.

## Conflict of Interest

The authors declare that the research was conducted in the absence of any commercial or financial relationships that could be construed as a potential conflict of interest.

## Publisher's Note

All claims expressed in this article are solely those of the authors and do not necessarily represent those of their affiliated organizations, or those of the publisher, the editors and the reviewers. Any product that may be evaluated in this article, or claim that may be made by its manufacturer, is not guaranteed or endorsed by the publisher.
